# High-dose-androgen-induced autophagic cell death to suppress the Enzalutamide-resistant prostate cancer growth via altering the circRNA-BCL2/miRNA-198/AMBRA1 signaling

**DOI:** 10.1038/s41420-022-00898-6

**Published:** 2022-03-22

**Authors:** Lei Chen, Yin Sun, Min Tang, Denglong Wu, Zhendong Xiang, Chi-Ping Huang, Bosen You, Dongdong Xie, Qinglin Ye, Dexin Yu, Chawnshang Chang

**Affiliations:** 1 Department of Urology, The Second Affiliated Hospital of Anhui Medical University, Hefei, Anhui 230000 China; 2 George Whipple Lab for Cancer Research, Departments of Pathology, Urology, Radiation Oncology and The Wilmot Cancer Institute, University of Rochester Medical Center, Rochester, NY 14642 USA; 3 Department of Urology, The First Affiliated Hospital of Nanjing Medical University, Nanjing, China; 4 Department of Urology, Tongji Hospital, School of Medicine, Tongji Universiry, Shanghai, 200092 China; 5 Sex Hormone Research Center, Department of Urology, China Medical University/Hospital, Taichung, 404 Taiwan, ROC

**Keywords:** Cancer prevention, Chaperone-mediated autophagy

## Abstract

Androgen deprivation therapy (ADT) is a gold standard treatment for advanced PCa. However, most patients eventually develop the castration-resistant prostate cancer (CRPC) that progresses rapidly despite ongoing systemic androgen deprivation. While early studies indicated that high physiological doses of androgens might suppress rather than promote PCa cell growth in some selective CRPC patients, the exact mechanism of this opposite effect remains unclear. Here we found that Enzalutamide-resistant (EnzR) CRPC cells can be suppressed by the high-dose-androgen (dihydrotestosterone, DHT). Mechanism dissection suggested that a high-dose-DHT can suppress the circular RNA-BCL2 (circRNA-BCL2) expression via transcriptional regulation of its host gene BCL2. The suppressed circRNA-BCL2 can then alter the expression of miRNA-198 to modulate the AMBRA1 expression *via* direct binding to the 3′UTR of AMBRA1 mRNA. The consequences of high-dose-DHT suppressed circRNA-BCL2/miRNA-198/AMBRA1 signaling likely result in induction of the autophagic cell death to suppress the EnzR CRPC cell growth. Preclinical studies using in vivo xenograft mouse models also demonstrated that AMBRA1-shRNA to suppress the autophagic cell death can weaken the effect of high-dose-DHT on EnzR CRPC tumors. Together, these in vitro and in vivo data provide new insights for understanding the mechanisms underlying high-dose-DHT suppression of the EnzR CRPC cell growth, supporting a potential therapy using high-dose-androgens to suppress CRPC progression in the future.

## Introduction

Prostate cancer (PCa) is the most frequently diagnosed cancer in American men and the sixth leading cause of cancer death in the world [1, 2]. Androgen deprivation therapy (ADT) is a gold standard treatment strategy for advanced PCa but castration-resistant prostate cancer (CRPC) following ADT has become a major obstacle [3, 4]. Enzalutamide (Enz), an androgen receptor-targeting agent, has shown significant anti-cancer effects in patients with CRPC and hormone sensitive PCa (HSPC) [5–8]. Unfortunately, when resistance to Enz develops [9, 10], the patients have poor prognoses and quality-of-life, and limited therapeutic options [11].

With an in-depth understanding of the biology of AR involvement in the CRPC process, the use of active AR signals to explore treatment opportunities has recently attracted increasing attention in the clinical setting [12–15]. Bipolar androgen therapy (BAT), which was pioneered by the Denmeade and Isaacs group is an emerging treatment for the inhibition of advanced PCa [16]. With BAT treatment, PCa patients received intermittent testosterone injections and experienced androgen levels between the supraphysiological and near-castrate range, and it was speculated that by rapid cycling of testosterone levels, the adaptive changes in AR expression will become dull, thereby delaying the appearance of resistance. At the same time, BAT likely improvs the quality- of -life [17, 18]. However, mechanisms underlying the suppression of PCa by the use of high doses of androgens are unclear. Several hypotheses have been proposed for the efficacy of bipolar therapy, including repression of SKP2 and MYC [19–22], induction of double-stranded DNA (dsDNA) breaks in conjunction with topoisomerase 2B [23, 24] and inhibition of DNA replication relicensing via stabilization of nuclear AR [18]. Understanding the underlying mechanisms of BAT will help design therapies to enhance their efficacy and provide precise treatment options for PCa patients.

Here we found high-dose-androgen may function via the circBCL2/miR-198/AMBRA1 signaling to induce autophagic cell death, providing new insights for the efficacy of high-dose-DHT to suppress the Enz resistant (EnzR) CRPC cell growth.

## Results

### A high-dose-DHT can suppress the EnzR CRPC cell growth

The paradoxical inhibitory response of PCa to supra-physiological androgens has been demonstrated with in vitro studies and in some selective subsets of patients. However, there is limited reports on whether a high-dose-DHT can also suppress CRPC at a later EnzR stage.

To test the efficacy of bipolar androgen therapy (BAT) in the two EnzR cell lines (EnzR1-C4-2 and EnzR4-C4-2B) generated by our Lab, we first cultured these EnzR cells in RPMI-1640 media supplied with 10 µM Enz and 2 nM DHT. This culture system has great advantages compared to the other culture system where PCa cells were cultured in charcoal/dextran-stripped fetal bovine serum (CS-FBS) media, which removed not only androgens but also other physiological important lipid molecules.

**Fig. 1:**
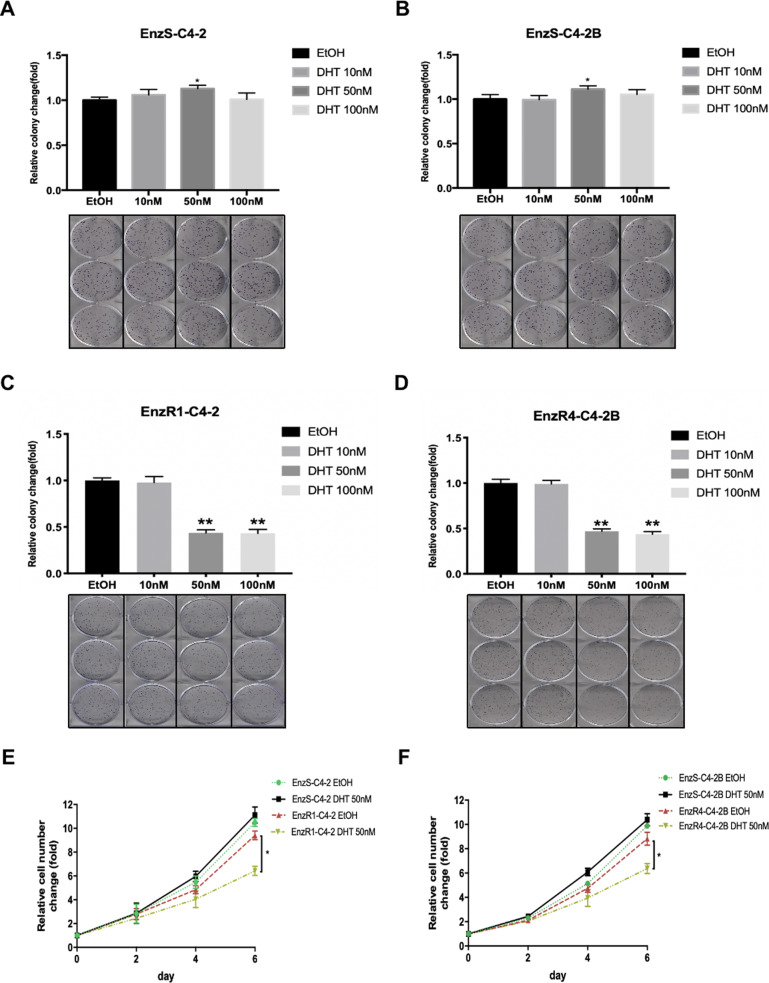
DHT can inhibit Enzalutamide-resistant PCa cells growth. **A**–**D** DHT could affect MTT assays, therefore colony formation assays were performed to show EnzS1-C4-2 (**A**) EnzS4-C4-2B (**B**), EnzR1-C4-2 (**C**), and EnzR4-C4-2 (**D**) cells growth with different concentrations of DHT. **E**, **F** Cell counting assays to show cell proliferation in EnzS vs EnzR cells (C4-2 and C4-2B) treated with EtOH or 50 nM DHT. Data are presented as means ± SD. *p < 0.05, **p < 0.01.

We then added different doses of DHT in the following two pairs of cell lines: EnzR1-C4-2 and EnzR4-C4-2B as well as their parental cells EnzS1-C4-2 and EnzS4-C4-2B, and assayed their impacts on the cell growth using colony formation assays. The results revealed that the cell growth was little changed at various concentrations of DHT for parental EnzS1-C4-2 and EnzS4-C4-2B cells. In contrast, we found the growth of EnzR1-C4-2 and EnzR4-C4-2B cells was significantly suppressed when adding DHT higher than 50 nM (Fig. 1A–D). Similar results were also obtained when we used direct counting of the cell number (Fig. 1E, F).

Together, results from Fig. 1A–F suggest high-dose-DHT can suppress the growth of EnzR CRPC cells.

### Mechanism dissection of how high-dose-DHT can suppress the EnzR CRPC cell growth: via inducing the autophagic cell death

**Fig. 2:**
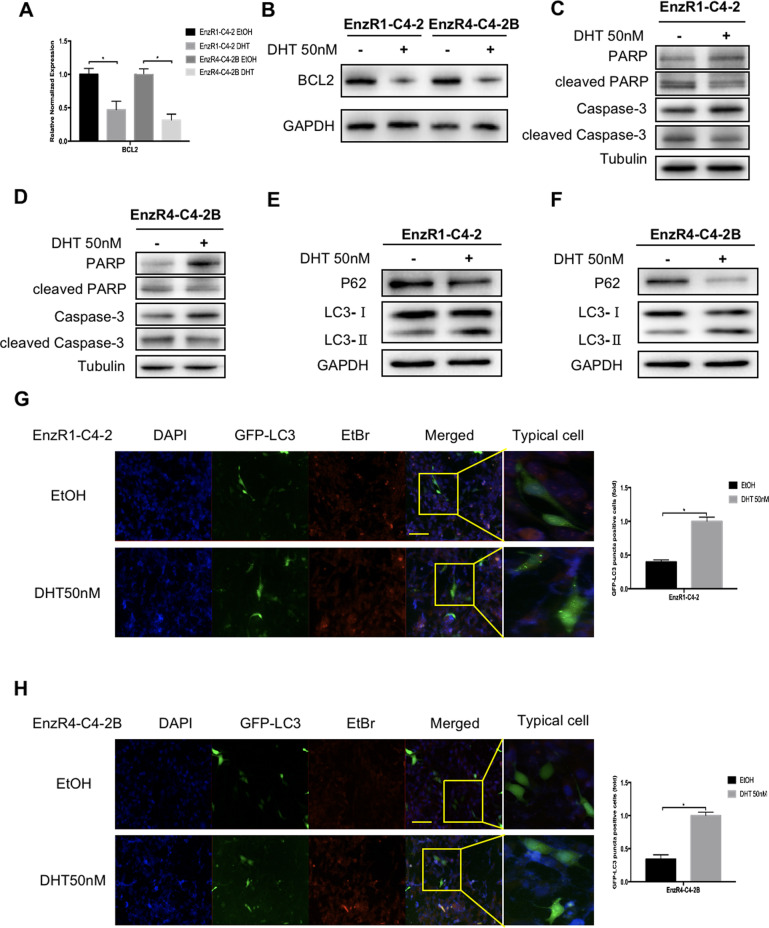
High-dose-DHT suppresses EnzR PCa cell growth via induction of autophagic cell death. **A** BCL2 mRNA expression in EnzR1-C4-2 and EnzR4-C4-2B cell lines treated with/without (w/wo) 50 nM DHT. **B** BCL2 protein expression in EnzR1-C4-2 and EnzR4-C4-2B cell lines treated w/wo 50 nM DHT. **C**, **D** Western blot assays were performed on EnzR1-C4-2 (**C**) and EnzR4-C4-2B (**D**) cell lines to show that caspase-dependent apoptosis marker is decreased with 50 nM DHT treatment. **E**, **F** Western Blot was performed on EnzR1-C4-2 and EnzR4-C4-2B cell lines to show that autophagy markers LC3 (LC3-I and LC3-II) are increased (**E**) and p62 is decreased (**F**) with 50 nM DHT treatment. **G**, **H** GFP-LC3 plasmid transfection was performed using Lipofectamine 3000 transfection reagent on EnzR1-C4-2 (**G**) and EnzR4-C4-2B (**H**) cells to show that GFP-LC3 puncta positive cells increased with 50 nM DHT treatment. The fluorescence images were captured by microscopy, scale bar = 40 µm. Data are presented as means ± SD. *p < 0.05.

As recent studies indicated that DHT could regulate apoptotic proteins [25, 26], we assayed potential impacts of high-dose-DHT on the cell death-related proteins, and results revealed that a high-dose-DHT (50 nM) could suppress the expression of the BCL2, an anti-apoptotic protein, at the mRNA (Fig. 2A) and protein levels (Fig. 2B) in the EnzR1-C4-2 and EnzR4-C4-2B cells, suggesting that high-dose-DHT may function via inducing apoptosis to suppress EnzR cell growth.

We then examined the expression of cellular apoptosis-related markers, including PARP1, cleaved PARP1, caspase, and cleaved caspase 3, to determine whether a conventional cell apoptosis or autophagic cell death is involved in the high-dose-DHT suppressed EnzR cell growth. The results revealed that adding high-dose-DHT led to decrease the expression of cleaved PARP1 and cleaved caspase 3 (Fig. 2C, D), as well as increase the expression of autophagy-related proteins, including LC3, and decrease the expression of p62/SQSTM1 in the EnzR1-C4-2 (Fig. 2E) and EnzR4-C4-2B (Fig. 2F) cells. Importantly, we also found that high-dose-DHT led to increase the GFP-conjugated LC3 puncta positive cells in EnzR1-C4-2 (Fig. 2G) and EnzR4-C4-2B (Fig. 2H) cells.

Together, results from Fig. 2A–H suggest that high-dose-DHT may function via inducing the autophagic cell death rather than Caspase-regulated apoptosis to suppress the EnzR cell growth.

Consistent with this conclusion, results from colony formation assays (see Fig. 1A–D) also indicated that the number of cell colony, and not the size of the cell colony, was significantly decreased after adding high-dose-DHT, supporting the conclusion that high-dose-DHT may increase cell death rather than suppress the cell proliferation/cell cycle of EnzR CRPC cells.

Together, results from Fig. 2A–H suggest that high-dose-DHT may suppress EnzR CRPC cell growth via inducing the autophagic cell death.

### Increasing BCL2 could not block, while increasing circRNA-BCL2 could reverse the high-dose-DHT suppression effect on the EnzR PCa cells growth

**Fig. 3:**
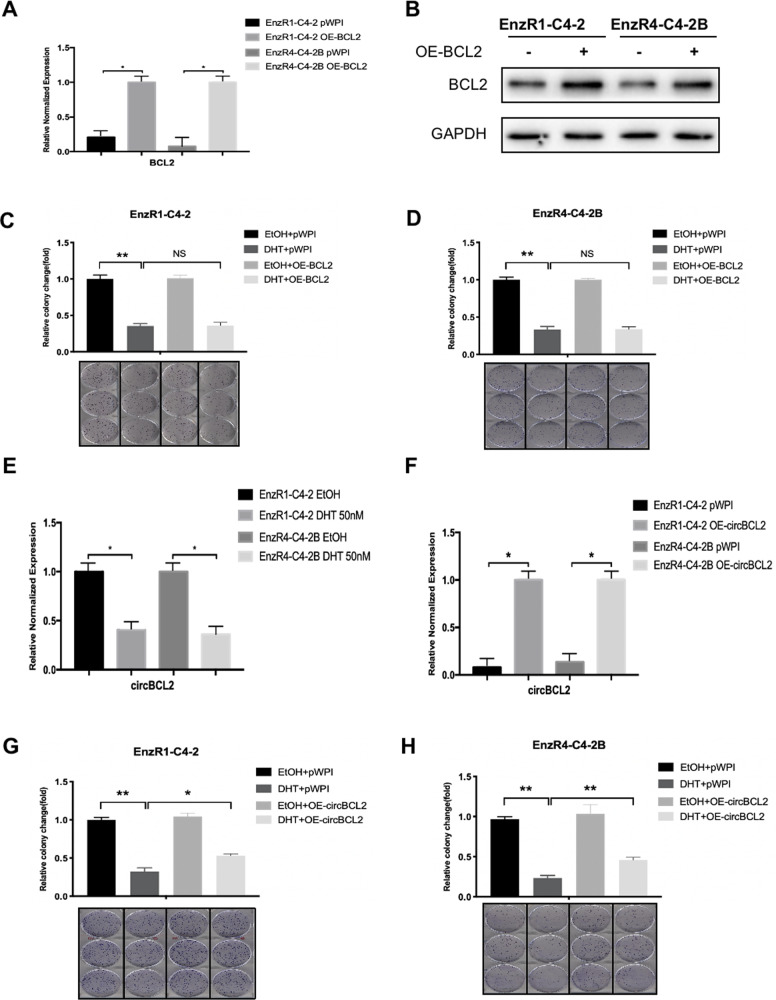
Increasing BCL2 could not block, while increasing circBCL2 could reverse, the DHT inhibition effect on the EnzR PCa cell growth. **A** The qRT-PCR was performed to show quantification of BCL2 overexpressing (OE) BCL2 (OE-BCL2) in EnzR1-C4-2 and EnzR4-C4-2B cell lines with/without (w/wo) OE-BCL2. **B** Western blot assays show BCL2 expression in EnzR1-C4-2 and EnzR4-C4-2B cell lines w/wo OE-BCL2. **C**, **D** Colony formation assays show cell growth with 50 nM DHT in EnzR1-C4-2 (**C**) and EnzR4-C4-2B (**D**) cells. **E** The circBCL2 expression in EnzR1-C4-2 and EnzR4-C4-2B cell lines with 50 nM DHT. **F** The qRT-PCR was performed to show quantification of circBCL2 w/wo OE-circBCL2 treated w/wo 50 nM DHT. **G**, **H** Colony formation assays show cell growth with 50 nM DHT in EnzR1-C4-2 and EnzR4-C4-2B cell lines w/wo OE-circBCL2. Data are presented as means ± SD. *p < 0.05, **p < 0.01, NS not significant.

To further test that high-dose-DHT may suppress EnzR cell growth *via* altering the BCL2 expression, we increased BCL2 expression via adding lentiviral BCL2-cDNA. The results revealed that increased BCL2 protein **(**Fig. 3A, B) led to little impact on the high-dose-DHT-suppressed cell growth using colony-forming assays in the EnzR1-C4-2 (Fig. 3C) and EnzR4-C4-2B cells (Fig. 3D).

We then focused on the circRNAs, a form of non-coding RNAs that have been increasingly recognized as being critical for all biological processes with distinct functions to influence development of several diseases including tumor progression [27, 28]. Results from a survey of BCL2 gene locus indicated that there is one circRNA associated with the BCL2 gene, (named as the circRNA-BCL2), and adding high-dose-DHT led to suppress circRNA-BCL2 expression (Fig. 3E).

Importantly, results from interruption assays revealed that overexpression of the circRNA-BCL2 (OE-circBCL2) with a lentiviral construct **(**Fig. 3F) led to partially reverse the high-dose-DHT-suppressed EnzR cell growth (Fig. 3G, H).

Together, results from Fig. 3A-H suggest that circBCL2, but not the BCL2 gene, can regulate the efficacy of high-dose-DHT through regulating circRNA-related biological activity, further supporting the view that autophagic cell death and not the conventional cell death is induced upon high-dose-DHT treatment.

### Mechanism dissection of how high-dose-DHT/circRNA-BCL2 signaling can induce autophagic cell death in the EnzR CRPC cells: via altering the AMBRA1 expression

It has been shown that androgens suppress BCL2 expression through negatively modulating the activities of the E2F site in the BCL2 promoter by activating the CDKI-RB axis [29, 30]. Consistent with that, we found that a high-dose-DHT could suppress the expression of the BCL2 at the mRNA and protein levels (see Fig. 2A, B), suggesting that a high-dose-DHT might suppress the circRNA-BCL2 expression via transcriptional regulation of its host gene BCL2.

**Fig. 4:**
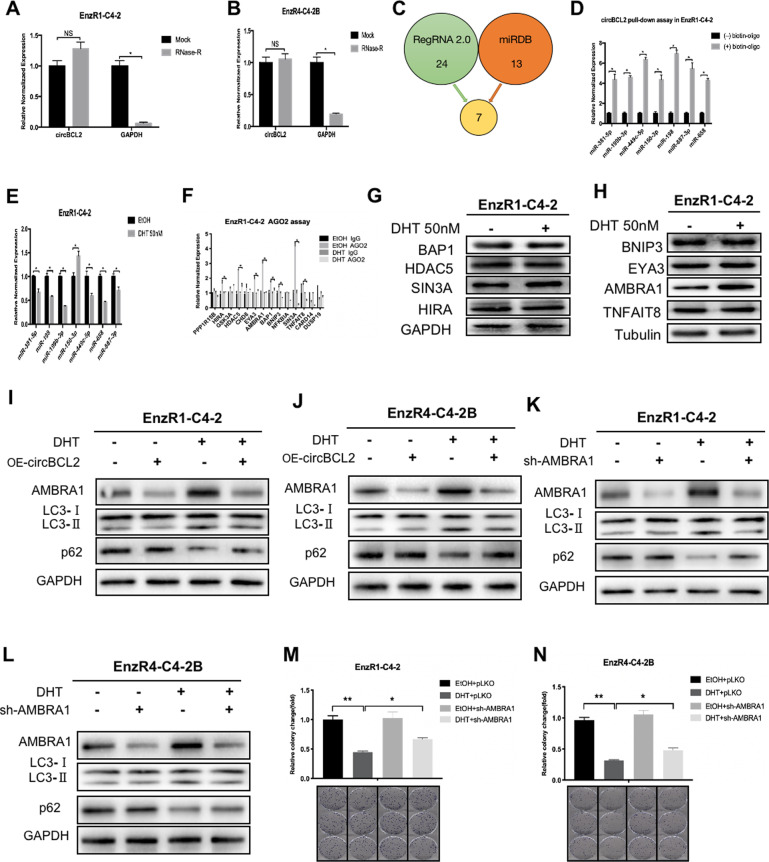
Mechanism dissection of how DHT/circRNA-BCL2 induces autophagic cell death in EnzR PCa cells: via regulating the AMBRA1 expression. **A**, **B** RNase R assay to determine the sensitivity of circBCL2 by RNase R digestion in EnzR1-C4-2 (**A**) and EnzR4-C4-2B (**B**) cell lines. **C** Venn diagram for prediction of the potential miRNAs using two miRNA related websites (miRDB and Regrna2.0). **D** Seven miRNA candidates can physically interact with circRNA-BCL2 and showed up after screening EnzR-C4-2 cells by biotin-circRNA-BCL2 pull-down assay. **E** The qRT-PCR was performed to show quantification of circRNA-BCL2 related miRNAs expression in EnzR-C4-2 cells after 50 nM DHT treatment. **F** Argonaute2 (AGO2) IP assay to detect 14 candidate genes mRNA in AGO2 complex and results revealed that 8 genes have less miRNAs binding in EnzR-C4-2 cells after 50 nM DHT treatment. **G**, **H** Western blot assays were performed to show candidates genes expression in EnzR-C4-2 cells after 50 nM DHT treatment. **I**, **J** Western blot assays show AMBRA1, LC3, and p62 expression after oe-circRNA-BCL2 in EnzR1-C4-2 (**I**) and EnzR4-C4-2B (**J**) cell line with 50 nM DHT. **K**, **L** Western blotting show AMBRA1, LC3 and p62 expression after sh-AMBRA1 in EnzR1-C4-2 (**K**) and EnzR4-C4-2B (**L**) cell line with 50 nM DHT. **M**, **N** Colony formation assays show cell growth with 50n M DHT when sh-AMBRA1 is transduced in EnzR1-C4-2 (**M**) and EnzR4-C4-2B (**N**) cell lines. Data are presented as means ± SD. *p < 0.05, **p < 0.01, NS not significant.

Next, we focused on how high-dose-DHT/circRNA-BCL2 signaling can induce autophagic cell death in the EnzR CRPC cells. Early studies indicated that circRNAs with intron sequences could regulate transcription while being more resistant to the exonucleases digest [31, 32]. We first confirmed that our identified circRNA-BCL2 is indeed a circRNA that is resistant to the RNase R digestion in EnzR1-C4-2 (Fig. 4A) and in EnzR4-C4-2B cells (Fig. 4B).

We then examined if circRNA-BCL2 may function via acting as a sponge for the miRNAs to impact the DHT effect, as this aspect of circRNA function has been found in many circumstances including those involved in breast or colorectal tumor progression [27]. Through bioinformatic prediction of miRNAs that can interact with circBCL2 (http://mirdb.org/ and http://regrna2.mbc.nctu.edu.tw) we identified 7 potential miRNAs with the numbering less than 1000 (Fig. 4C). Results from the pull-down experiment with biotin-conjugated antisense oligonucleotide for the junction sequence of circRNA found that all of them appeared to be able to bind to the circBCL2 (Fig. 4D). We also examined their expression in response to high-dose-DHT treatment and found 6 of 7 had decreased expression after treating with the high-dose-DHT (Fig. 4E).

To identify the potential common target genes that can be regulated by these miRNAs alone or in combination, we searched their target genes by testing if those genes have the potential to have a more significant regulation as a result of being targeted by all those miRNAs simultaneously. We further manually curated the candidate target genes based on their likely involvement in cell cycle, signal transduction, apoptosis, autophagy, and gene transcription, and identified a list of 14 genes that can be potentially targeted by these miRNAs. To further select the likely target genes regulated by these miRNAs, we performed an AGO2 RIP assay and the results revealed that 8 of them could be regulated by miRNAs in response to DHT treatment (Fig. 4F). Western blot analysis with lysates prepared from cells treated with and without DHT indicated that AMBRA1 changed significantly after DHT (50 nM) treatment (Fig. 4G, H), suggesting that AMBRA1 is likely a direct target for the DHT initiated signaling, consistent with our earlier findings that autophagic cell death was likely mediating the growth suppression by DHT.

To directly implicate AMBRA1 in DHT-mediated growth suppression, we used western blot analysis to confirm that adding DHT resulted in AMBRA1 increase in both CRPC cell lines (Fig. 4I, J). Importantly, decreased AMBRA1 expression via adding AMBRA1-shRNA led to partially reverse/block the effect of high-dose-DHT on the decrease of the LC3 (LC3-I and LC3-II) and increase of the p62 in the EnzR1-C4-2 (Fig. 4K) and EnzR4-C4-2B cells (Fig. 4L). Similar results were shown with colony formation assays (Fig. 4M, N).

Together, results from Fig. 4A–N suggest that high-dose-DHT/circRNA-BCL2 signaling may induce autophagic cell death via regulating the AMBRA1 expression in the EnzR CRPC cells.

### Mechanism dissection of how DHT/circRNA-BCL2 signaling can alter the AMBRA1 expression in the EnzR CRPC cells: via regulating the miRNA-198 expression

**Fig. 5:**
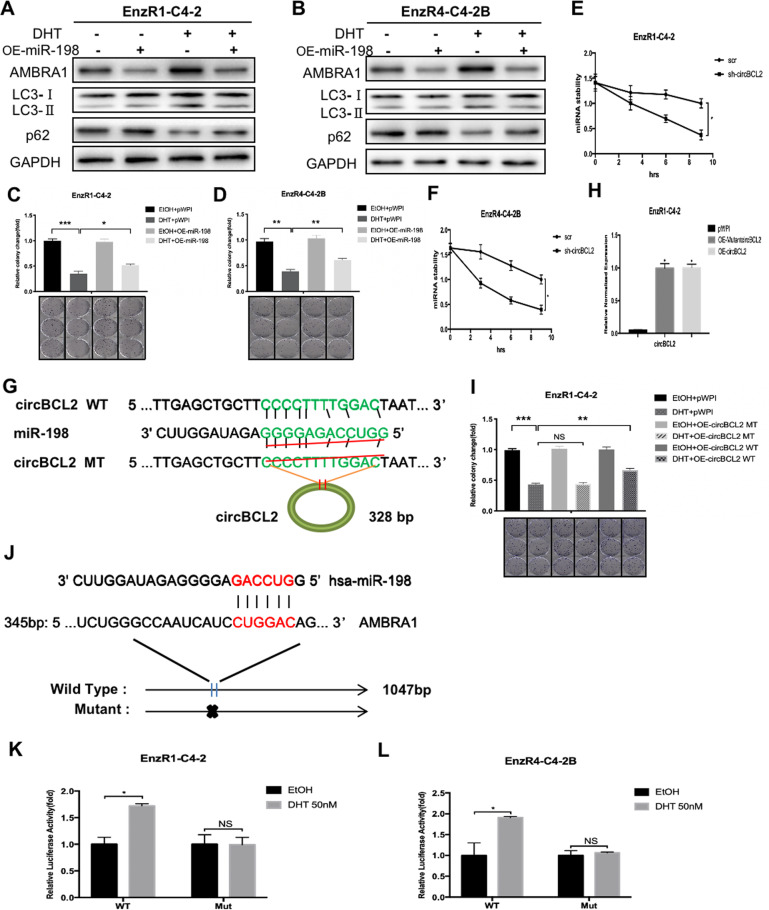
Mechanism dissection of how DHT/circRNA-BCL2 alters the AMBRA1 expression in EnzR PCa cells: via regulating the miRNA-198 expression. **A**, **B** Western blot assays show AMBRA1, LC3 and p62 expression after OE-miRNA-198 (OE-miR-198) transduced in EnzR1-C4-2 (**A**) and EnzR4-C4-2B (**B**) cell lines with 50 nM DHT. **C**, **D** Colony formation assays show cell growth with 50 nM DHT when OE-miR-198 transduced in EnzR1-C4-2 (**C**) and EnzR4-C4-2B (**D**) cell lines. **E**, **F** The miR-198 stability was measured with the addition of 5 μM Actinomycin D (ActD) in EnzR1-C4-2 (**E**) and EnzR4-C4-2B (**F**) cells with/without knocked-down circRNA-BCL2. **G** The circRNA-BCL2 with and without miR-198 binding sites (CircNet). **H** The qRT-PCR was performed to show quantification of circRNA-BCL2 after OE-mutantcircBCL2. **I** Colony formation assay shows decreased cell growth with 50 nM DHT and OE-mutant circRNA-BCL2 in EnzR1-C4-2 cell line. **J** The wild type (WT) and mutant (Mut) AMBRA1 luciferase constructs. **K**, **L** Luciferase activity after transfection of WT or Mut AMBRA1 3’-UTR with 50 nM DHT treatment in EnzR1-C4-2 (**K**) and EnzR4-C4-2B (**L**) cell lines. Data are presented as means ± SD. *p < 0.05, **p < 0.01, ***p < 0.001, NS not significant.

Next, to dissect the molecular mechanism of how DHT/circRNA-BCL2 signaling can function via sponging the miRNAs or serve as a reservoir of miRNAs to alter the AMBRA1 expression, we focused on miRNA-658, miRNA-199b-3p, and miRNA-198 as these 3 can target AMBRA1 based on bioinformatic analysis. We tested whether their expression could reverse the high-dose-DHT effects on the AMBRA1 expression. Results from western blot analysis and colony formation assays suggested that miRNA-198 is the most consistent candidate that could partially reverse the effects of the high-dose-DHT (Fig. 5A–D, Fig. S1A, B) in both EnzR cell lines.

In addition, we found the circRNA-BCL2 expression was positively correlated with the miRNA-198 expression, showing the stability of miR-198 was reduced when circRNA-BCL2 was silenced, with a consequent decrease of its expression level in the EnzR1-C4-2 and EnzR4-C4-2B cells (Fig. 5E, F), suggesting that circBCL2 might function as a reservoir to keep miRNA-198 more stable, instead of serving as a sponge to inactivate its function.

To further demonstrate that circ-BCL2 may function via stabilizing miRNA-198 to mediate the high-dose DHT effects to suppress EnzR cell growth, we also generated a mutant circRNA-BCL2 deficient in binding to miRNA-198 (Fig. 5G), and results using colony-forming assays revealed that adding mutant circRNA-BCL2, and not wild-type circ-BCL2, failed to reverse/block the high-dose-DHT effects to suppress the cell growth in the EnzR1-C4-2 and EnzR4-C4-2B cells (Fig. 5H, I).

Together, results from Fig. 5A–I suggest that DHT/circRNA-BCL2 signaling can alter the AMBRA1 expression *via* regulating the miRNA-198 expression in the EnzR CRPC cells.

### Mechanism dissection of how high-dose-DHT/circRNA-BCL2/miRNA-198 signaling can alter the AMBRA1 expression in the EnzR CRPC cells: via direct binding to the 3′UTR of AMBRA1 mRNA

To further study the molecular details of how high-dose-DHT/circRNA-BCL2/miRNA-198 signaling can alter the AMBRA1 expression, we identified potential miRNA binding sites that matched the seed sequence of miRNA-198 on the 3′UTR of AMBRA1 mRNA with subsequent construction of the reporter plasmids using the psiCheck2 vector carrying the wild-type and mutant miRNA-target sites (Fig. 5J). As expected, the luciferase assay results revealed that adding high-dose-DHT markedly increased luciferase activity in EnzR1-C4-2 (Fig. 5K). and EnzR4-C4-2B cells (Fig. 5L) transfected with wild-type 3’UTR of AMBRA1 mRNA, but not the mutant 3’UTR AMBRA1 mRNA.

Together, results from Fig. 5J–L suggest high-dose-DHT/circRNA-BCL2/miRNA-198 signaling can alter the AMBRA1 expression via direct binding to the 3′UTR of AMBRA1 mRNA.

### Preclinical study using the in vivo xenograft mouse model to confirm the role of DHT and AMBRA1 in EnzR cell growth

**Fig. 6:**
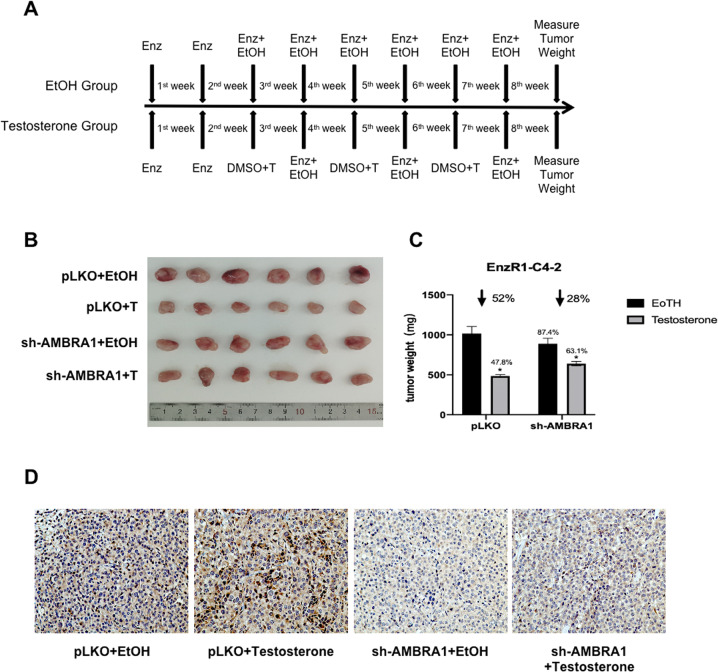
Preclinical study using In vivo mouse model to confirm the role of DHT and AMBRA1 in EnzR cell growth. **A** Equal numbers of EnzR1-C4-2 cells (5 × 10^6^ cells) were subcutaneously injected into each mouse to establish the CRPC xenograft model. Mice were injected with Enz (10 mg/kg/week, twice weekly). Bipolar androgen therapy treatment was conducted by injection of Testosterone (200 μg/kg/week, twice weekly) or EtOH at the 3th/5th/7th week. Mice were euthanized after 8 weeks and tumors were removed for studies. **B**, **C** Tumor weights (**B**) were shown and presented as means ± SD (**C**). **D** Representative IHC images of AMBRA1 expression in tumor tissue samples from the 4 groups, ×200 magnification. *p < 0.05.

To test whether suppression of AMBRA1 can block the DHT’s suppressive effect on PCa tumor growth in a xenograft model in vivo, we generated cells with stable expression of sh-AMBRA1 as well as vector control (PLKO) cells that were subcutaneously transplanted into nude mice with following treatment options, (1) pLKO + EtOH, (2) pLKO + testosterone, (3) sh-AMBRA1 + EtOH, and (4) sh-AMBRA1 + testosterone (Fig. 6A). After 8 weeks of treatment, we found that the pLKO + testosterone group had the smallest tumor size (Fig. 6C, D), while sh-AMBRA1 led to a reduced effect of testosterone on suppressing tumor growth by DHT.

**Fig. 7:**
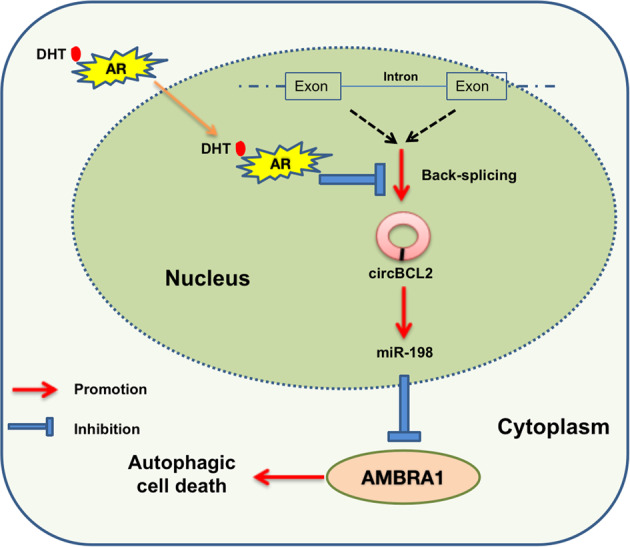
A mechanistic diagram. A mechanistic diagram for DHT effect in CRPC cells.

Together, these in vivo results demonstrated that a lower AMBRA1 expression could block the inhibitory effect of high-dose-DHT (Fig. 7).

## Discussion

For the past 70 years, blocking androgen signaling has been the focus of treatment strategies for advanced and metastatic PCa [33]. First-line ADT, either through surgical or medical castration, has brought hope to PCa patients, however, the disease inevitably develops into castration resistance [34]. Treatment with Enz, a second-generation ADT, can reduce the risk of metastasis or death by 71% and extend patients survival an extra 3–5 months for non-metastatic CRPC patients [35–37]. But most of them will eventually develop Enz-resistance with limited therapeutic options. Therefore great efforts have focused on inhibiting the growth of CRPC through maintaining castration-equivalent androgen levels while blocking other molecular targets along the androgen signaling axis. At the same time, parallel projects have utilized supraphysiologic androgen doses to exploit unique susceptibilities within CRPC cells and subsequently delay disease progression. This approach was first utilized with modest success in murine xenografts [18, 38]. With increasing understanding of the biology and AR involvement in the CRPC process, some novel approaches to treat patients with CRPC have been developed, including bipolar androgen therapy (BAT).

BAT, a therapeutic approach in which supraphysiologic doses of testosterone are delivered at regular intervals, only to be followed by a rapid clearance of testosterone as androgen levels are once again reduced to levels consistent with castration [23, 39, 40]. Recently, its efficacy has been confirmed in some clinical trials [41, 42]. The mechanism of high-dose-androgen inhibiting PCa is still unclear. Several hypotheses have been proposed for the efficacy of BAT. First, the AR-axis stimulated by supraphysiologic DHT can promote the formation of double-strand breaks (dsDNA) through recruiting AR-driven enzymes to common vulnerable sites in the genome that are prone to abnormal rearrangements [43]. Another mechanism proposed involves the co-recruitment of AR and TOP2B to sites of TMPRSS2-ERG genomic break-points, facilitating formation of transient DSB secondary to TOP2B catalytic cleavage [40]. Third, androgen-activated AR serves as a licensing factor for DNA replication to block cell proliferation [44]. More recently it was shown that non-chromatin binding of AR might contribute to the high-dose-DHT effect through transcriptional co-activator redistribution [45].

The circRNAs are non-coding RNAs with high conservation and abundance and form closed continuous loops from exons or introns by back splicing or lariat formation [46, 47]. Early studies demonstrated that a circRNA can function as an endogenous, competing miRNA “sponge” to absorb and degrade miRNAs, thus affect disease progression [48]. Here, we provided additional regulation of circRNA on miRNAs through a circRNA serving as a stabilizing factor for miRNA expression, opposite to the sponging ability to suppress miRNA function (Fig. 5E, F). This novel function of circRNA was demonstrated through cell line studies showing the congruent function of circRNA-BCL2 and miRNA-198 in suppressing AMBRA1 expression. Mechanistically, we can speculate the binding of miR-198 with circRNA-BCL2 might enhance the miR-198 stability by repressing its degradation through nucleases such as Tudor-SN endonuclease. The exact details of these phenomena remain to be further determined.

In this work, we characterize the pro-autophagic protein AMBRA1. It is primarily expressed in neural tissues and is essential for normal neural tube development [49]. As a scaffold protein, AMBRA1 fine tunes autophagy by bringing together proteins functionally related to the autophagy pathway [50, 51]. More recently, it was found that AMBRA1 is a component of the bona fide E3 ligase for Cyclin D, thus a direct connection to cell cycle regulation [52–54]. On the other hand, previous studies have demonstrated that AMBRA1 is a positive factor for autophagy induction, mainly through interaction with mTORC1, ULK1, Beclin1, dynein light chain1/2 (DLC1/2), and BCL2 located at the mitochondria (mito-BCL2) [55, 56]. Considering AMBRA1’s place in the cascade of events leading to autophagy regulation, we propose that AMBRA1 plays a key role in this context. Indeed, we find that a high concentration of androgen can suppress Enz-resistant PCa cells through the induction of autophagic cell death as a result of increased AMBRA1 expression. This enhanced AMBRA1 expression is also in line with its role in degrading Cyclin D to suppress cell proliferation, consistent with previous findings that high-dose-DHT can suppress cell proliferation. In support of these roles of AMBRA1, downregulation of AMBRA1 with shRNA in EnzR PCa cells made the cells’ survival more resistant to the effect of increased testosterone in vivo.

In summary, our study reveals that high-dose-DHT could inhibit circRNA-BCL2 expression and decrease miRNA-198 expression, thus inducing AMBRA1 expression with consequent triggering of autophagic cell death. These studies provide a novel mechanism for how high-dose-DHT inhibits CRPC cell growth and potentially new therapeutic approaches to enhance bipolar androgen therapy for CRPC patients.

## Materials and methods

### Cell culture

Human PCa cell lines C4-2 (EnzS-C4-2) and 293T cells were obtained from the American Type Culture collection (ATCC, Rockville, MD). RPMI 1640 and DMEM were used to culture these PCa cells and 293T cell, respectively. All PCa cells were cultured in RPMI 1640 (without phenol red) supplied with 2 nM DHT,10% fetal bovine serum (FBS), penicillin (25 U/ml) and streptomycin (25 mg/ml) in the humidified 5% CO_2_ environment at 37 °C.

### Generation of Enz-resistant cell lines

C4-2R (EnzR1-C4-2) cells were generated by culturing C4-2 (EnzS-C4-2) cells under increasing Enz concentrations from 10 μM to 40 μM (every 20 days) for three months. C4-2B (EnzS-C4-2B) and C4-2BR (EnzR4-C4-2B) cells were gifts from Dr. Allen Gao (University of California, Davis, USA). All EnzR cells were maintained in media with 10 µM Enz.

### Lentiviral infection

The plasmids pLKO.1-AMBRA1, pWPI-circBCL2, pWPI-mutantcircBCL2, pLKO.1-miR-198, the psPAX2 packaging plasmid, and pMD2.G envelope plasmid, were transfected into HEK-293T cells using the standard calcium chloride transfection method for 48 h to get the lentivirus soup. The lentivirus soups were collected and concentrated by density gradient centrifugation and used immediately or frozen at −80 °C for later use.

### RNA extraction, miRNA extraction, and qRT-PCR analysis (qRT-PCR)

For RNA extraction, Trizol reagent (Invitrogen) was used to isolate total RNAs and 1 µg of total RNA was subjected to reverse transcription into cDNA using Superscript III transcriptase (Invitrogen). Determination of mRNA expression level of a gene of interest was completed using quantitative real-time PCR (qRT-PCR) conducted using a Bio-Rad CFX96 system with SYBR green. The expression of GAPDH was used to normalize the expression levels of a target gene.

For measurement of miRNAs, briefly, 1 μg of total RNA was processed with poly A polymerase at 37 °C for 20 min, and then annealing at 65 °C for 5 min, 4 °C for 2 min after adding 50 μm RT anchor primer. The last step was cDNA synthesis at 42 °C for 60 min adding 2 μl 10 mM dNTP, 2 μl 5 × RT buffer, 1 μl reverse transcriptase and ddH2O to a total of 20 μl. The qRT-pCR protocol was conducted as 95 °C 2 min, followed by 45 cycles at 95 °C 15 s, 60 °C 45 s. U6 and/or RPL32 were used as normalization.

### Western blot analysis

Cells were lysed in RIPA buffer and proteins (30 µg) were separated on 8–10% SDS/PAGE gel and then transferred onto PVDF membranes (Millipore). After blocking membranes, they were incubated with appropriate dilutions of specific primary antibodies, and then blots were incubated with HRP-conjugated secondary antibodies and visualized using ECL system (Thermo Fisher Scientific).

### Tests of RNase R resistance

Cell total RNAs were isolated by TRIZOL followed by Pure Link purification of the aqueous phase (Life Technologies). Total RNA at 1 µg was treated with 20 U RNase R (Epicenter) or mock in 1× RNase R buffer in a 10 μl reaction volume with 1 U/μl Ribonuclease Inhibitor (New England Biolabs), then incubated at 37 °C for 1 h. Then 1 μl of 10 mM dNTP, 1 μl of 1 mM EDTA, and 1 μl of 100 M random hexamer were added and mixture denatured at 65 °C for 5 min, then placed on ice. Four μl 5x buffer (250 mM Tris-HCl/pH 8.0, 125 mM KCl, 15 mM MgCl2), 1 μl murine Ribonuclease Inhibitor [40U/μl], and 1 μl Superscript III (LifeTechnologies) were added. This cDNA reaction procedure was 25 °C 10 min, 50 °C 50 min, 55 °C 10 min, 85 °C 5 min, and then held at 4 °C. One μl cDNA reaction was used as the template for qPCR.

### Colony formation assay

EnzR1-C4-2 and EnzR4-C4-2B cells were plated in 6-well plates containing 200–500 cells in each well and allowed to grow for an additional 10–14 days. Then, the culture solution was discarded and plates carefully rinsed twice with PBS. Cells were then fixed using 4% paraformaldehyde for 20 min and then stained with 0.5% crystal violet staining solution for 20 min. The colonies containing more than 50 cells were counted under a microscope.

### Pull-down assay

The cultured cells were collected and lysed in RIPA lysis buffer. The supernatants were mixed with 500 pM of anti-sense oligos supplemented with RNAase inhibitor over-night at 4 °C. The cells were mixed for 2 h at 4 °C after adding 10 μl Streptavidin Agarose beads, then the streptavidin Agarose beads were incubated with the supernatant for 2 h. The complex was centrifuged at 3000 rpm for 10 min, and the beads were washed five times with RIPA lysis buffer. The RNA was extracted using Trizol (Invitrogen) according to the manufacturer’s protocol and subjected to qRT-PCR analysis.

### GFP-LC3 plasmid transfection and confocal microscopy

A total of 2 × 10^4^ cells were plated on coverslips for 24 h. GFP-LC3 plasmid transfection was performed using Lipofectamine 3000 transfection reagent (Invitrogen, L3000) according to the manufacturer’s protocol. After a 24 h incubation, the media containing the transfection mixture was replaced with fresh complete media and the cells were treated with/without DHT (50 nM) for 48 h. Then the cells were fixed in 4% formaldehyde for 20 mins, and incubated with DAPI and EtBr. The LC3 puncta were examined with a confocal micro-scope (TSC-SP8, Leica, Germany). For each group, three independent images were randomly selected to count the number of LC3 puncta positive cells.

### Luciferase reporter assay

The AMBRA1 3’UTR with wild-type (WT) or mutant (Mut) miRNA-responsive elements was cloned into the psiCHECK-2 vector (Promega) downstream of the Renilla luciferase ORF. EnzR1-C4-2 and EnzR4-C4-2B cells were plated in 24-well plates and the plasmides were transfected with Lipofectamine 3000 transfection reagent (Invitrogen, Carlsbad, CA) according to the manufacturer’s instructions. DHT was added 24 h after transfection Luciferase activity was measured 48 h after transfection by Dual-Luciferase Assay (Promega) according to the manufacturer’s manual.

### In vivo studies

All the animal experiments were performed in accordance with the guidelines for the care and use of laboratory animals and were approved by the Medical Center Animal Care and Use Committee of Anhui Medical University. Male Balb/c Nude mice at 6 weeks old were purchased from Cavens (Changzhou, China) and divided into 4 groups for injection of 5 × 10^6^ EnzR1-C4-2 cells that were pre-cultured with PLKO or sh-AMBRA1 and treated as follows: (1) PLKO + EtOH, (2) PLKO + Testosterone, (3) sh-AMBRA1 + EtOH, and (4) sh-AMBRA1 + Testosterone. Enz was given to all mice through intraperitoneal injection (10 mg/kg/week, twice weekly). Bipolar androgen therapy treatment was conducted by injected administration of Testosterone (200 µg/kg/week, twice weekly) or EtOH at the 3rd/5th/7th week. Mice were sacrificed after 8 weeks.

### H&E and immunohistochemical (IHC) staining

Paraffin-embedded tissue sections (4.0 µm) were deparaffinized, dehydrated, and rehydrated in a graded ethanol series. Three percent H_2_O_2_ was added to block endogenous peroxidase activity. After a rinse in distilled water and phosphate-buffered saline (PBS) successively, sections were placed in citrate buffer (10 mM/pH 6.0) and heated in a microwave oven at 95 °C for 30 min. Then, the sections were incubated with primary antibody (Anti-FLJ20294, 1:200, Abclonal) at room temperature for 1.5 h and 4 °C over-night. The color reaction was developed with the HRP-linked polymer detection system (SP9002, ZSGB-BIO, Beijing, China) and counterstaining with hematoxylin. Images were captured with an Axio Scope A1 microscope (Carl Zeiss, Germany) and analyzed using the ZEN Blue Lite software (Carl Zeiss, Germany).

### Statistics

All statistical analyses were carried out with SPSS 19.0 (SPSS Inc, Chicago, IL). The results were representative of experiments repeated at least three times with data points in triplicate and presented as the mean ± SD. Differences in mean values between two groups were analyzed by two-tailed Student’s *t* test and the mean values of more than two groups were compared with one-way ANOVA. Statistical significance was determined when *p* value was ≤0.05 (*p* ≤ 0.05).

## Supplementary information

Supplementary Information file

## Data Availability

All the data are available in the article and Supplementary Files, or available from the authors upon request.
