# Supplementary material for: High-dose-androgen-induced autophagic cell death to suppress the Enzalutamide-resistant prostate cancer growth via altering the circRNA-BCL2/miRNA-198/AMBRA1 signaling
Source: Cell Death Discov. 2022 Mar 22;8:128. doi: 10.1038/s41420-022-00898-6 (PMC8941094; doi:10.1038/s41420-022-00898-6)
Supplement: Supplementary file 1 — Supplementary Information file [file 41420_2022_898_MOESM1_ESM.pdf]

western blots

Figure 2B bcl2

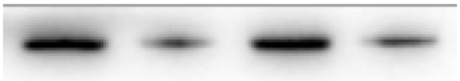

Figure 2B GAPDH

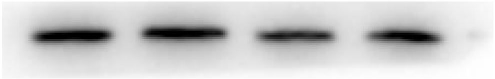

Figure 2C caspase3

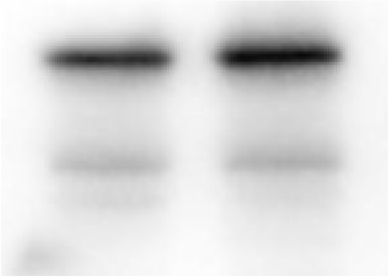

Figure 2C cleaved caspase3

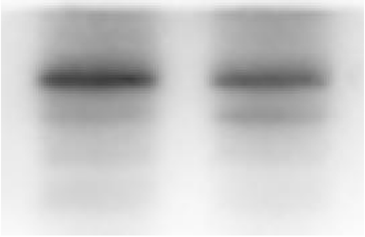

Figure 2C PARP

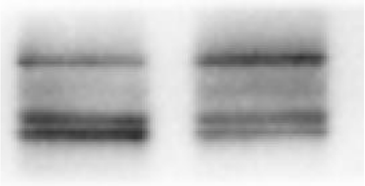

Figure 2C tubulin

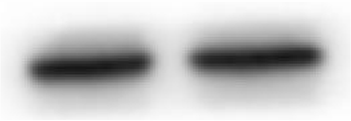

Figure 2D caspase3

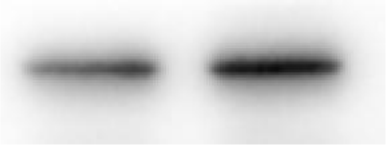

Figure 2D cleaved caspase3

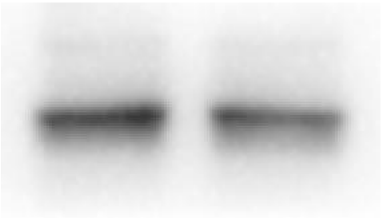

Figure 2D PARP

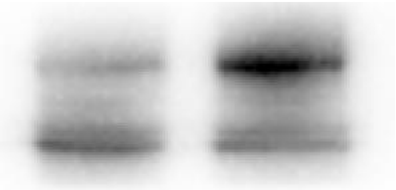

Figure 2D tubulin

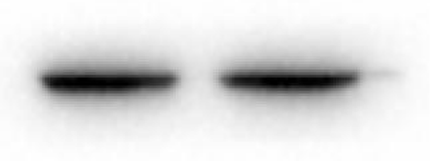

Figure 2E GAPDH

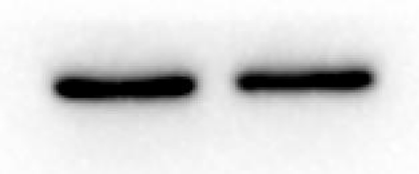

Figure 2E LC3

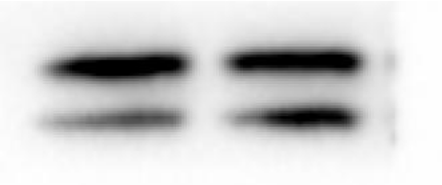

Figure 2E p62

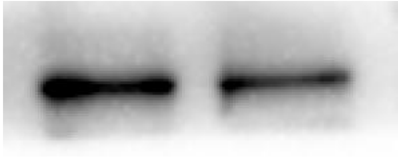

Figure 2F GAPDH

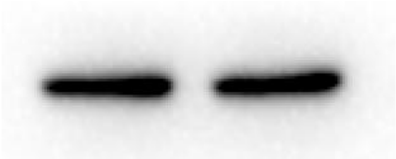

Figure 2F LC3

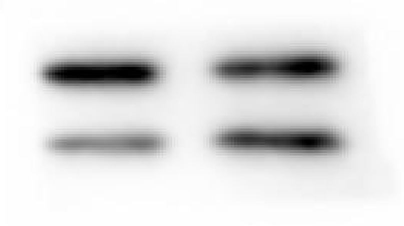

Figure 2F p62

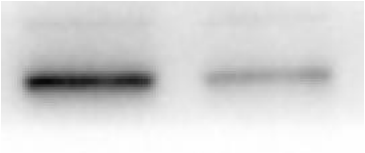

Figure 3B BCL-2

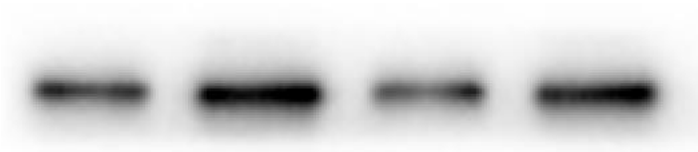

Figure 3B GAPDH

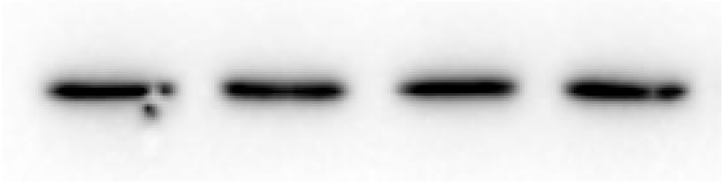

Figure 4G BAP1

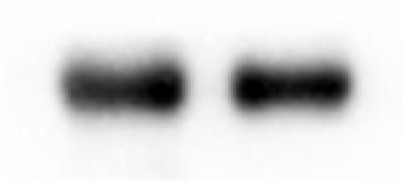

Figure 4G GAPDH

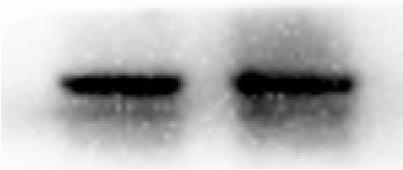

Figure 4G HDAC5

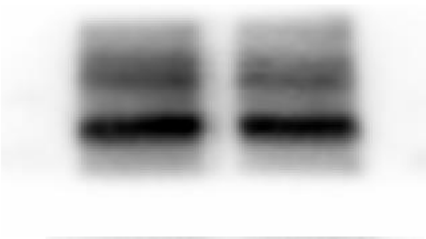

Figure 4G HIRA

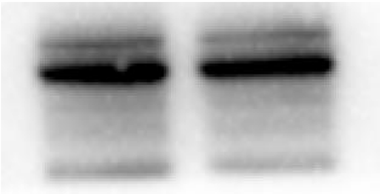

Figure 4G SIN3A

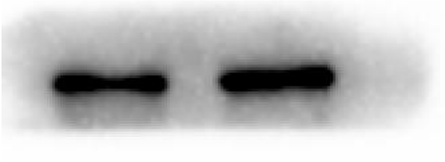

Figure 4H AMBRA1

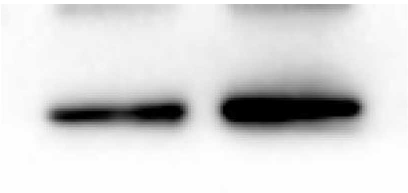

Figure 4H BNIP3

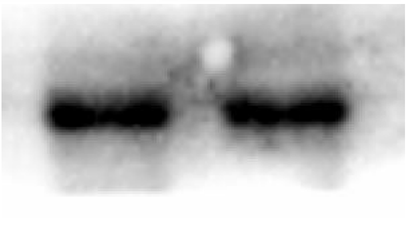

Figure 4H EYA3

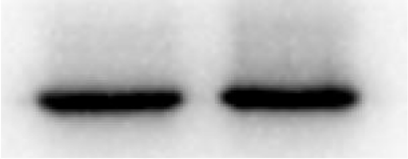

Figure 4H TNFAIT8

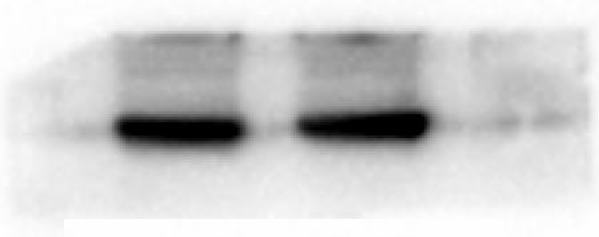

Figure 4H TUBULIN

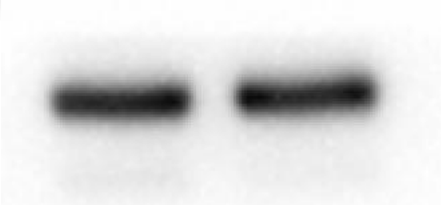

Figure 4I AMBRA1

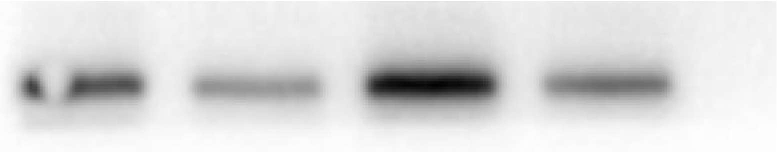

Figure 4I GAPDH

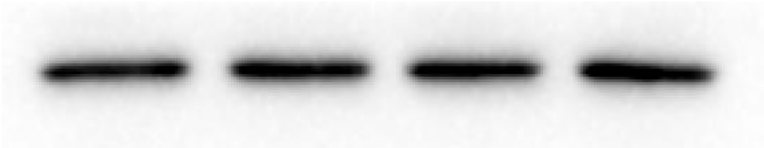

Figure 4I LC3

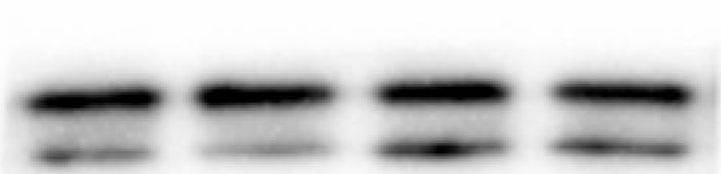

Figure 4I p62

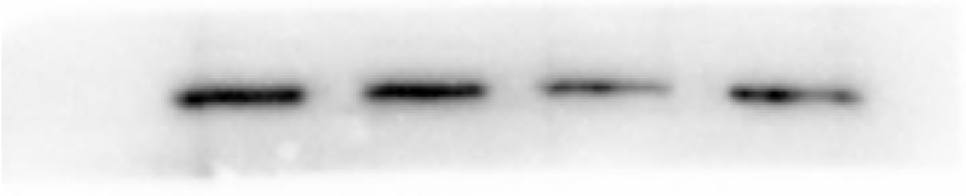

Figure 4J AMBRA1

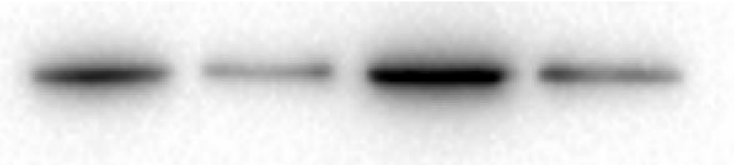

Figure 4J GAPDH

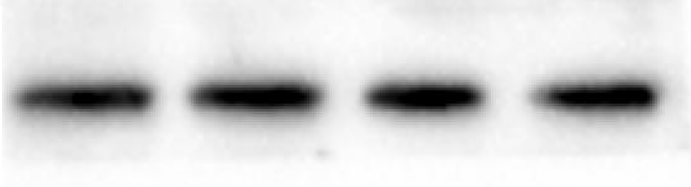

Figure 4J LC3

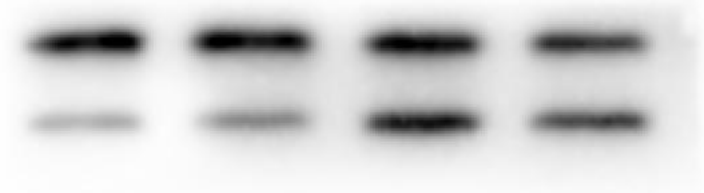

Figure 4J P62

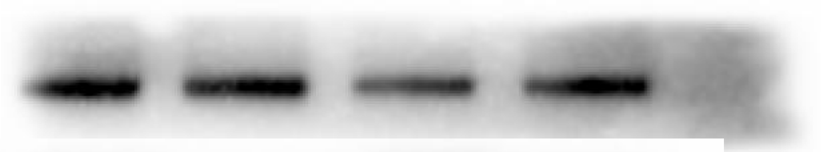

Figure 4K AMBRA1

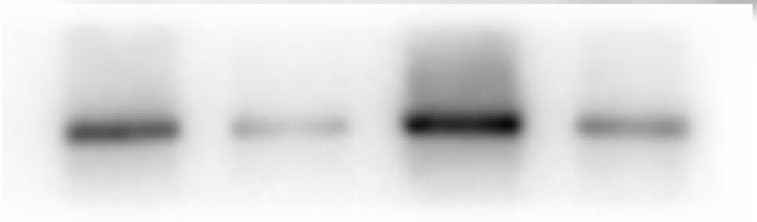

Figure 4K GAPDH

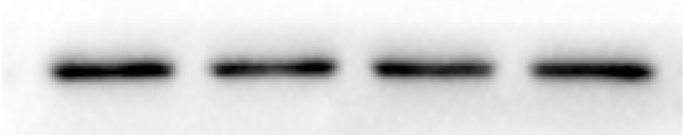

Figure 4K LC3

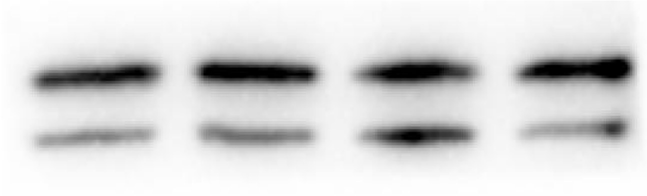

Figure 4K P62

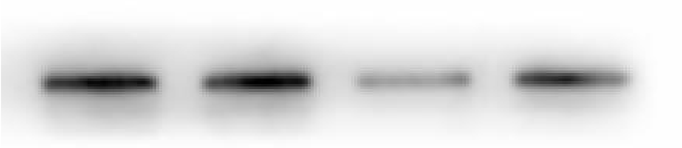

Figure 4L AMBRA1

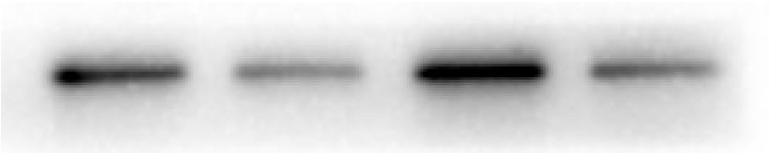

Figure 4L GAPDH

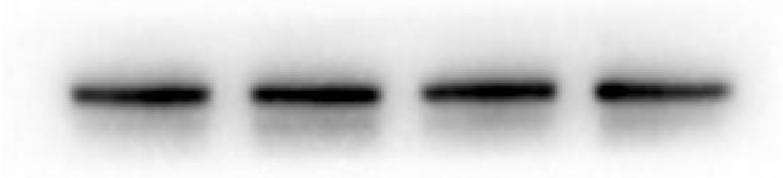

Figure 4L LC3

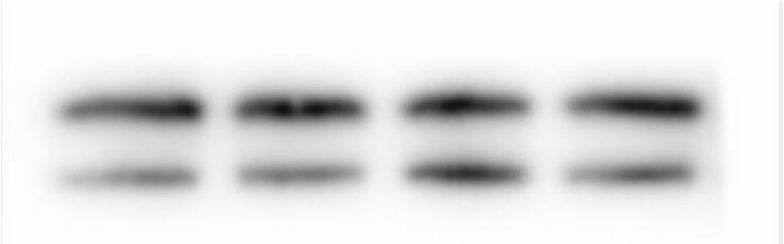

Figure 4L P62

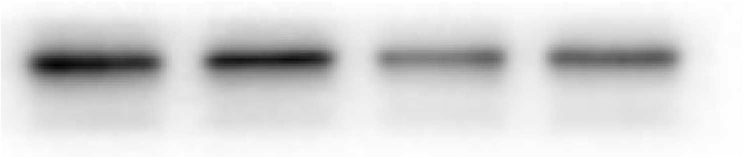

Figure 5A AMBRA1

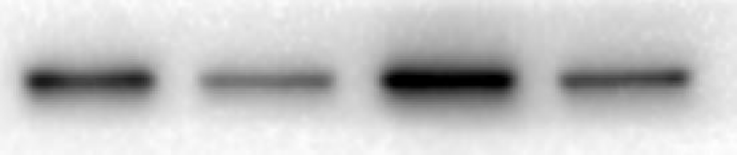

Figure 5A GAPDH

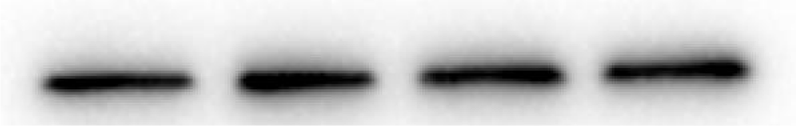

Figure 5A LC3

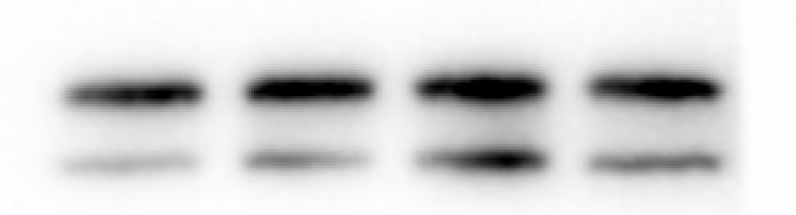

Figure 5A P62

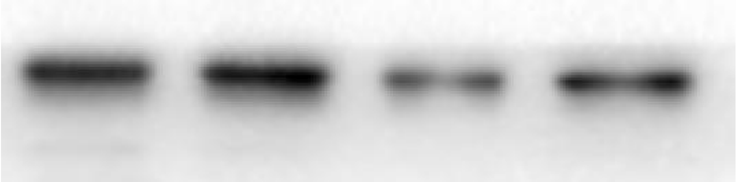

Figure 5B AMBRA1

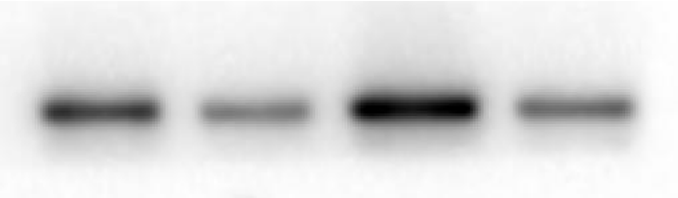

Figure 5B GAPDH

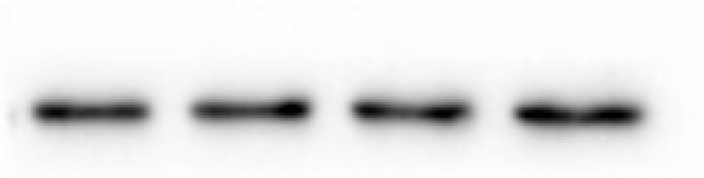

Figure 5B LC3

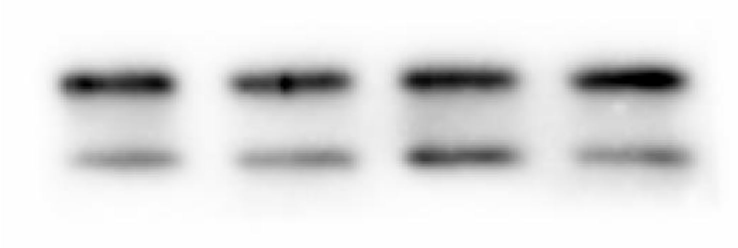

Figure 5B P62

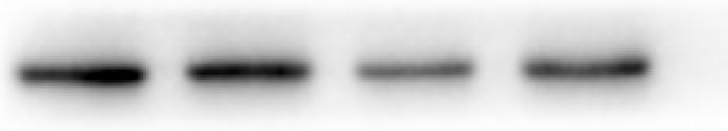

Figure.S1

A

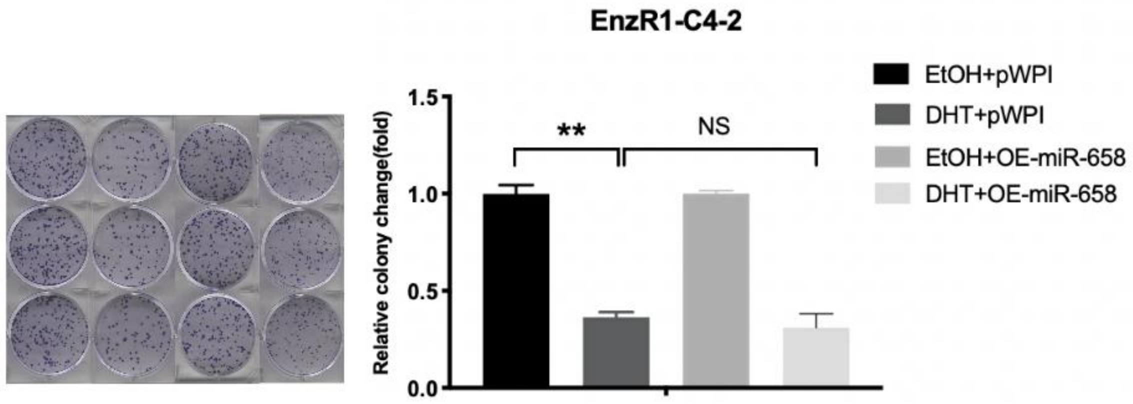
EnzR1-C4-2
EtOH+pWPI
DHT+pWPI
EtOH+OE-miR-658
DHT+OE-miR-658
Relative colony change(fold)
1.5
1.0
0.5
0.0
**
NS

B

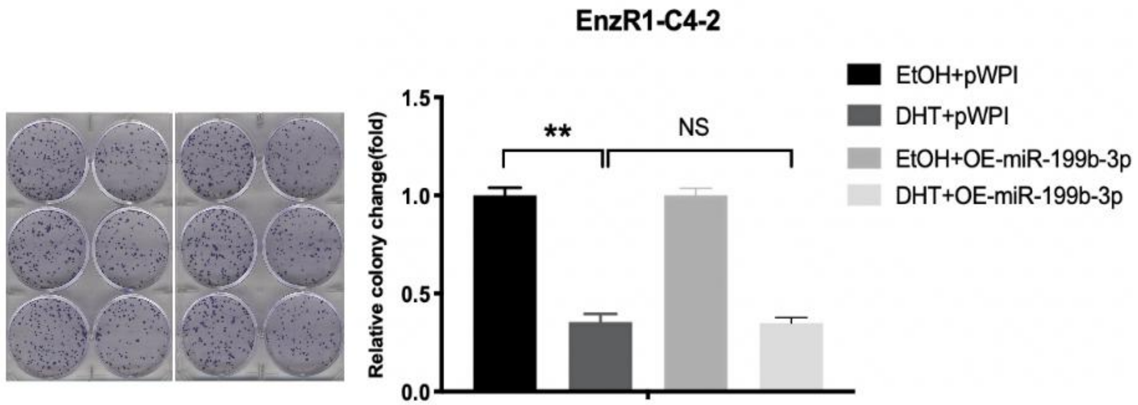
EnzR1-C4-2
EtOH+pWPI
DHT+pWPI
EtOH+OE-miR-199b-3p
DHT+OE-miR-199b-3p
Relative colony change(fold)
1.5
1.0
0.5
0.0
**
NS

plasmid list

1.oe-mutant-circBCL2:
ACAGAAACTCAGGATCTGTTCAACAGTAATGCCTGTTCCCC
CACGCCTGCAGCCTTGGCAACCACCATACTACTGCCTGCCT
CTATGAACTTGACTACTCTAGGTACCTCGCATAAGTGGACTC
ACACTTGTGACTGGCTTGTTACACTAAGCATGATGTCTTCAA
GGTTCATCCATGTTTTGGCCTGTGTCACAGCTTCCTTCATTT
TGAAGGCTGAATGATATTCCATTGAATGGATCTACTACATAT
TGTTTCTCCGTTCATTTATTGATGGATACTTGAGCTGCTTTAA
TGTGAATAATGCTGCTATGAACG

2.oe-circBCL2:
ACAGAAACTCAGGATCTGTTCAACAGTAATGCCTGTTCCCC
CACGCCTGCAGCCTTGGCAACCACCATACTACTGCCTGCCT
CTATGAACTTGACTACTCTAGGTACCTCGCATAAGTGGACTC
ACACTTGTGACTGGCTTGTTACACTAAGCATGATGTCTTCAA
GGTTCATCCATGTTTTGGCCTGTGTCACAGCTTCCTTCATTT
TGAAGGCTGAATGATATTCCATTGAATGGATCTACTACATAT
TGTTTCTCCGTTCATTTATTGATGGATACTTGAGCTGCTTCC
CCTTTTGGACTAATGTGAATAATGCTGCTATGAACG

3.oe-miRNA-198:
GTCCAGAGGGGAGATAGGT

4.psi-wildtype-AMBRA1:
AGACAAACGTTGCACTGGTGCCTCCCCTCGAACCGCCAAGC
AGAAACCGGACCTCACAGCTGACTGGGAACTGGACATGTGG
AAGAGCTGCTGGCTGCATCAGGGAACAGGAGGAGGAAGAG
GGTCAGGGTGGAGAGGAAGATCAGTCAGTGGGCACAAGAC
AGTCAAATGGGCAAGGCCTGCCTCGGGGAACTAGAACCTTC
CAGGATCTGGAGCCCGGGAGAGCCACACTGTGGGCTTAATG
TGAATAGAGGAACAAGTGGGTATCTCTGCCAGGCACCCCAC
TTTCTCCTAGTAACATGGGCTCAGGGGACTCAGCCCTGGAC
AGAGAGCCTCCAGAGAGTtctagaGAACAGTCTTCCAGATCTG
GGCCAATCATCCTGGACAGAGGCCCGCGAGGCAGCTTTGC
CCTGTCCACCTGTTGGGTGGGCAGAGCCACCAGGAACCCA
GACACCACCTCCAACTCTGAGCCTTCCAGAGCTTCAGCCTCT
CTTCGTCGTCTTACCCCACTGAAACCAACAGGGATCGGGCC
AGGCTCCCAGATTCTTGAGGACAGGGACTTCGGCATTTACTA
ATGGGGGACTACTGTGGGGTAAGGGGGCGCCTGCTTGCCT
GATACAGGATGGGGTCAAGGGACAGTGGGCAGGTCCTCACT
CAGGAGTGGGGGGTGTAGGCTGGCCAGCCCCCAGGGCTTG
TCCACCAGTCTTCTCCCCGCAAGGCCCTCAGAGCAGCGCCT
GTGGGTGTCAGTATTACCTGAGCCTAGGCCAAAGCTAGCCC
AAGGCTGGGGAAGGGGAGGAGACTCCAGGTCAGAATGTGA
GGTCTCAGTCTGTGATTTAAGGTGTTGCATGTGGACTCTTAA
CTGTACGTGTAGTTTCTAGTGGAGAAATCAAGGCTCTGATCA
TTTTGTTTTTAGTATGAAAATGTGATTTCCTTTCTGTTTGTAAC
TCATCATAGAAACATTGTGGTGGGAGGAGAGGGGATAGTCT
ACAGCTAATGAGGGAAACACCAAAGATCACATCATTAAAATG
ATGACATGCCCCTC

5.psi-mutant-AMBRA1:
AGACAAACGTTGCACTGGTGCCTCCCCTCGAACCGCCAAG
CAGAAACCGGACCTCACAGCTGACTGGGAACTGGACATGT
GGAAGAGCTGCTGGCTGCATCAGGGAACAGGAGGAGGAAG
AGGGTCAGGGTGGAGAGGAAGATCAGTCAGTGGGCACAAG
ACAGTCAAATGGGCAAGGCCTGCCTCGGGGAACTAGAACC
TTCCAGGATCTGGAGCCCGGGAGAGCCACACTGTGGGCTT
AATGTGAATAGAGGAACAAGTGGGTATCTCTGCCAGGCACC
CCACTTTCTCCTAGTAACATGGGCTCAGGGGACTCAGCCCT
GGACAGAGAGCCTCCAGAGAGTtctagaGAGGCCCGCGAGGC
AGCTTTGCCCTGTCCACCTGTTGGGTGGGCAGAGCCACCA
GGAACCCAGACACCACCTCCAACTCTGAGCCTTCCAGAGCT
TCAGCCTCTCTTCGTCGTCTTACCCCACTGAAACCAACAGG
GATCGGGCCAGGCTCCCAGATTCTTGAGGACAGGGACTTC
GGCATTTACTAATGGGGGACTACTGTGGGGTAAGGGGGCG
CCTGCTTGCCTGATACAGGATGGGGTCAAGGGACAGTGGG
CAGGTCCTCACTCAGGAGTGGGGGGTGTAGGCTGGCCAGC
CCCCAGGGCTTGTCCACCAGTCTTCTCCCCGCAAGGCCCT
CAGAGCAGCGCCTGTGGGTGTCAGTATTACCTGAGCCTAG
GCCAAAGCTAGCCCAAGGCTGGGGAAGGGGAGGAGACTCC
AGGTCAGAATGTGAGGTCTCAGTCTGTGATTTAAGGTGTTG
CATGTGGACTCTTAACTGTACGTGTAGTTTCTAGTGGAGAAA
TCAAGGCTCTGATCATTTTGTTTTTAGTATGAAAATGTGATTT
CCTTTCTGTTTGTAACTCATCATAGAAACATTGTGGTGGGAG
GAGAGGGGATAGTCTACAGCTAATGAGGGAAACACCAAAGA
TCACATCATTAAAATGATGACATGCCCCTC

6.sh-circBCL2:
PLKO-GCTATGAACGACAGAAACTCA

7.sh-AMBRA1:
PLKO-GGACACTACTTACTCACAGCAATTG
